# Supplementary figures and images for: Open and Closed Triple Inhaler Therapy in Patients with Uncontrolled Asthma
Source: Adv Respir Med. 2023 Jul 4;91(4):288–300. doi: 10.3390/arm91040023 (PMC10366885; doi:10.3390/arm91040023)

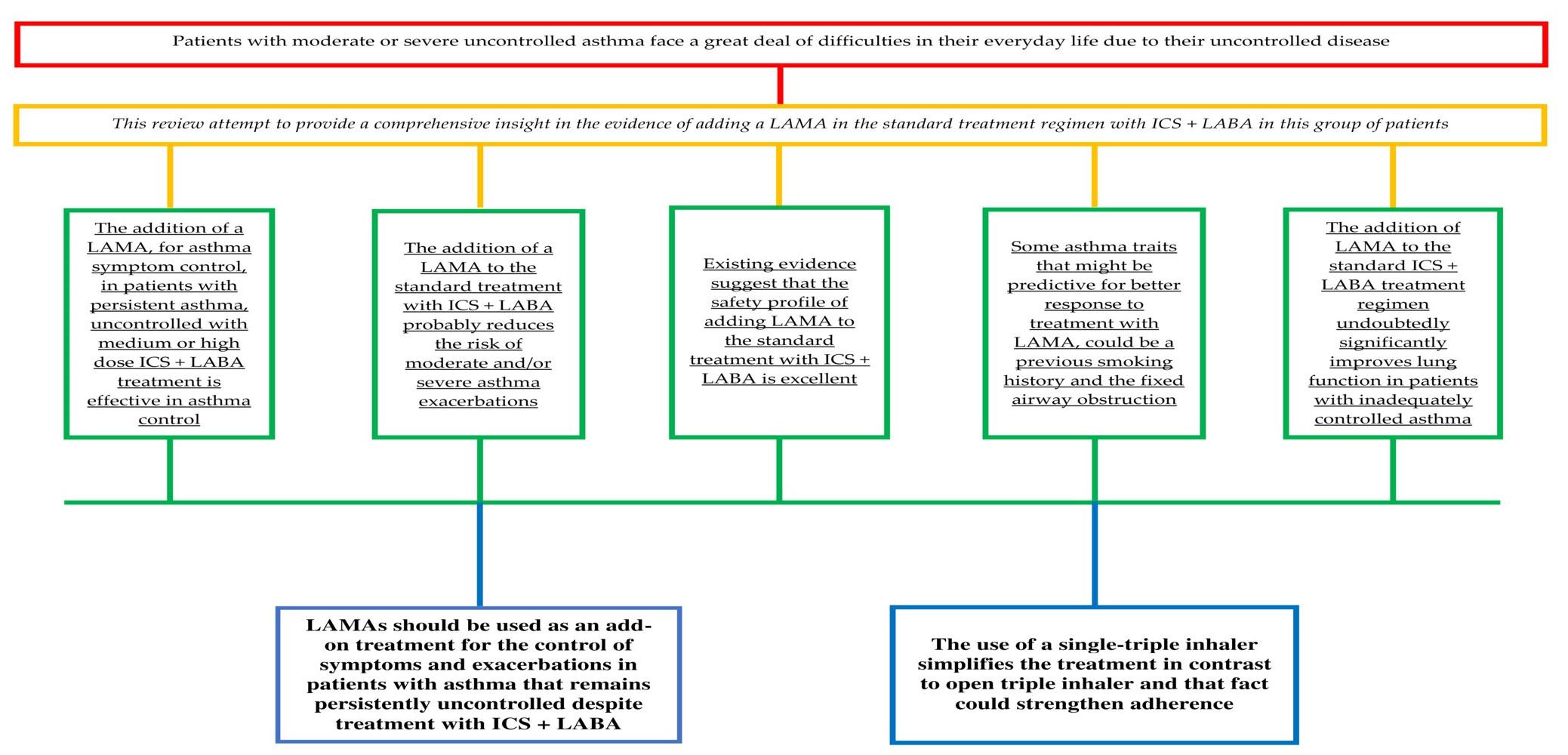

Supplement: Supplementary file 1 [file arm-91-00023-s001.zip › arm-2399938-supplementary Figure S1.jpg]
